# Risk of Malignancy with Immunosuppressive Drugs Used in Organ Transplants Compared to Those Used for Non-Transplant Indications

**DOI:** 10.3390/cancers18111784

**Published:** 2026-05-29

**Authors:** Connor Haines, Zachary Walton, Ian Curnutt, George Golovko, Yong-Fang Kuo, Cristiana Rastellini, Luca Cicalese

**Affiliations:** 1 John Sealy School of Medicine, University of Texas Medical Branch, Galveston, TX 77555, USA; cohaines@utmb.edu (C.H.); zcwalton@utmb.edu (Z.W.); 2 College of Osteopathic Medicine, Kansas City University, Joplin, MO 64804, USA; ian.curnutt@kansascity.edu; 3 Department of Biostatistics and Data Science, University of Texas Medical Branch, Galveston, TX 77555, USA; gegolovk@utmb.edu (G.G.); yokuo@utmb.edu (Y.-F.K.); 4 Department of Neurosciences, John Sealy School of Medicine, University of Texas Medical Branch, Galveston, TX 77555, USA; crrastel@utmb.edu; 5 Division of Transplant Surgery, Department of Surgery, University of Texas Medical Branch, Galveston, TX 77555, USA

**Keywords:** transplant, immunosuppressants, malignancy, neoplasia, cell-mediated, receptor-mediated, non-transplant

## Abstract

**Background:** Immunosuppressive drugs (ISDs) are essential for preventing organ rejection but have been reported to increase cancer risk with prolonged use. This study compares cancer risk between ISDs used for long-term maintenance after transplantation (T-ISDs) and those prescribed for non-transplant chronic conditions including cell-mediated (C-ISDs) and receptor-mediated (R-ISDs) ISDs. We hypothesized that cancer risk would differ between T-ISDs and both C-ISD and R-ISD groups. **Methods:** Using the TriNetX database, solid organ transplant recipients treated with tacrolimus (TAC), cyclosporine (CY), rapamycin (RAPA), or mycophenolate (MMF) were compared to propensity-matched R-ISDs (adalimumab, infliximab, etc.) or C-ISDs (methotrexate, azathioprine, etc.) for at least 24 encounters to determine risk of malignancy. Hazard ratios (HRs) with 95% confidence intervals (CIs) were calculated to assess the three-year cancer risk. **Results:** After matching, T-ISDs were associated with higher malignancy risk compared to both R-ISDs (n = 29,748; HR 2.616, 95% CI 2.427–2.820) and C-ISDs (n = 31,704; HR 1.271, 95% CI 1.195–1.351). Each individual immunosuppressant in the T-ISD cohort was associated with increased cancer risk compared to R-ISDs, while only TAC and CY showed higher risk than C-ISDs (TAC: n = 9846, HR 1.354, 95% CI 1.228–1.492; CY: n = 1801, HR 1.234, 95% CI 1.007–1.512). Organ-specific analyses showed consistent patterns across systems. **Conclusions:** Overall, T-ISDs are associated with increased malignancy risk compared to R-ISDs and modestly compared to C-ISDs. TAC and CY confer the greatest risk, while MMF demonstrates relatively lower relative risk. These findings underscore the need to individualize ISD regimens to minimize long-term cancer risk.

## Simple Summary

This retrospective cohort study used the TriNetX database to evaluate and compare cancer risk among patients receiving different classes of immunosuppressive drugs. The aim was to determine whether transplant-related ISDs are associated with a higher malignancy risk than ISDs used for non-transplant chronic conditions. Transplant-related ISDs were associated with a higher three-year cancer risk compared to both receptor-mediated and cell-mediated therapies, with the greatest difference seen versus receptor-mediated drugs. Tacrolimus and cyclosporine showed the highest risk, while mycophenolate had relatively lower risk. Overall, transplant-related immunosuppression carries a greater malignancy risk, emphasizing the need to individualize treatment strategies to reduce long-term cancer risk.

## 1. Introduction

In 1949, Hench et al. discovered the use of cortisone’s immunosuppressive properties in treating rheumatoid arthritis [1]. This discovery marked the beginning of the development of immunosuppressive drugs (ISDs) [1]. Following cortisone’s discovery, ISDs’ immunosuppressive abilities were applied to malignancy therapy, resulting in the development of cell-mediated therapies including methotrexate, cyclophosphamide, and 6-mercaptopurine. Schwartz and Dameshek in 1960 discovered that the immunosuppressive properties of chemotherapeutics drugs, namely 6-mercaptopurine, had the ability to delay skin graft rejection [2,3]. In 1963, Murray et al. used 6-mercaptopurine during a human kidney transplant to suppress rejection [4]. After these discoveries, tacrolimus (TAC), cyclosporin (CY), rapamycin (RAPA), and mycophenolate mofetil (MMF) targeting T and B lymphocytes were developed for long-term use in preventing transplant rejection and have been the most used drugs used in transplantation since. These transplant immunosuppressive drugs (T-ISDs) are commonly used in combination or independently.

In the 21st century, receptor-mediated immunosuppressants (R-ISDs) such as adalimumab, infliximab, and etanercept were introduced to target specific immune pathways in autoimmune diseases like rheumatoid arthritis and Crohn’s disease. These antibody-based drugs selectively inhibit molecules like TNF-α or IL-2 and offer more precise therapy, though their cost may limit access in certain healthcare settings or for uninsured populations [5,6]. Despite studies being performed to assess the effectiveness of the previously mentioned T-ISDs and R-ISDs, immunosuppressant treatment plans for transplants and chronic inflammatory diseases vary widely between patients due to the treating physician’s experience, preference, and the patient’s tolerance of these medications. A standardized ISD combination regimen has not been established because large-scale, long-term comparative data is lacking.

Cell-mediated immunosuppressants (C-ISDs) and R-ISDs (monoclonal or polyclonal antibodies and fusion proteins) are integral to current practices in autoimmune disease and chronic inflammatory disease management. In transplant patients, long-term administration of ISDs is critical for preventing graft rejection, preserving organ viability, and supporting overall well-being [7,8,9]. These therapies are also used in autoimmune conditions such as systemic lupus erythematosus, rheumatoid arthritis, and myasthenia gravis, where they help control aberrant immune activity and alleviate disease symptoms [10,11,12]. Despite their benefits, all ISDs impair immune surveillance, increasing vulnerability to bacterial, fungal, and viral infections. Individuals using ISDs chronically are susceptible to infections such as reactivated latent tuberculosis and hepatitis B and C [13]. These infections are not only harmful in themselves but are also associated with an increased risk of malignancy. Among organ transplant recipients, due to the potent and pronged use, the risk of virus-associated cancers has been reported to increase for Epstein–Barr virus-associated lymphomas, human papillomavirus-related cervical cancer, human herpesvirus-related Kaposi’s sarcoma, and chronic hepatitis B or C infection-related hepatocellular carcinoma [14,15,16].

Patients receiving long-term ISD therapy following organ transplantation face a heightened risk of developing malignancies, even those unrelated to viral infections [17]. Reported associations include cancers of the brain (notably lymphomas), cervix, and prostate [18,19,20]. While existing research evaluates the impact of specific ISDs or combinations, few studies directly compare malignancy risk in T-ISDs used for long-term post-transplant treatment to ISDs of differing mechanisms of action (cell- or receptor-mediated) used in non-transplant settings. Additionally, many of these studies are limited by small sample sizes and incomplete analyses of long-term risks. Moreover, we have recently shown that even limited ISD exposure may contribute to cancer development [21].

The increasing use of ISDs in treating non-transplant-related conditions such as systemic lupus erythematosus, Crohn’s disease, ulcerative colitis, myasthenia gravis, and other autoimmune diseases highlights the need to further investigate the associated risk of malignancy. While the therapeutic benefits of ISDs are well established, relatively few studies have explored the long-term cancer risks in patient populations with individual use of different ISDs. Notably, previous studies have reported that chronic TAC exposure following liver transplant correlates with increased recurrence of hepatocellular carcinoma [22]. However, the drug-specific malignancy risk profile in patients using cell-mediated T-ISDs like TAC, CY, MMF and RAPA and that of patients using ISDs for non-transplant-related conditions is underexplored and warrants further attention.

Although all ISDs contribute to immunosuppression, the degree to which individual agents or combinations elevate malignancy risk is not well understood. In clinical transplant practice, immunosuppressive regimens are typically administered as combination therapy and are frequently adjusted over time using therapeutic drug monitoring to balance efficacy and toxicity [23]. Calcineurin inhibitor minimization and individualized dose titration, often in combination with adjunctive agents such as mTOR inhibitors, are key components of long-term management strategies [24,25]. However, this study focuses on the independent effects of individual immunosuppressive agents on malignancy risk, rather than combination regimens or dose-dependent strategies.

Much of the current literature relies on transplant-specific datasets and often compares recipients to healthy controls, which is an approach that limits insight into drug-specific effects. Moreover, studies frequently report cumulative immunosuppression without directly contrasting individual drugs or multi-drug regimens. To address these gaps, we utilized TriNetX, an international health research platform that aggregates depersonalized patient data and allows for adjustment of comorbidities and confounding variables.

This study evaluates the cancer-related outcomes associated with three distinct classes of ISDs: T-ISDs, C-ISDs, and newer R-ISDs therapies. Prior studies have categorized immunosuppressive therapies into those primarily used in transplantation (T-ISDs) and those prescribed for non-transplant conditions (C-ISDs and R-ISDs) [26]. This distinction helps clarify how differences in mechanisms of action influence malignancy risk, allowing for a more precise evaluation of how specific agents modulate immune suppression and contribute to cancer development in transplant versus non-transplant populations.

## 2. Materials and Methods

We used the US Collaborative Network from 86 US healthcare organizations in the TriNetX Research Network. The available data included information about demographics; diagnoses coded in the International Classification of Diseases, Tenth Revision (ICD-10); procedures coded in the Current Procedural Terminology (CPT); and medications coded in Veterans Affairs National Formulary (VA). The network used by the TriNetX database contains patient records from 122,604,040 patients. Due to the anonymous nature of the data, informed consent was waived. The data was accessed in February 2025.

Three analytic cohorts were constructed based on the ISD of interest and the primary clinical indication for its use. Immunosuppressive agents routinely prescribed for long-term post-transplant maintenance including TAC, CY, MMF, and RAPA were assigned to the T-ISD cohort (Table 1). Cell-mediated medications used predominantly for immunosuppression in chronic autoimmune or inflammatory diseases unrelated to transplantation were categorized as the C-ISD cohort. This group comprised methotrexate, azathioprine, cyclophosphamide, hydroxyurea, capecitabine, and leflunomide (Table 1). Receptor-mediated immunosuppressants not routinely used in transplantation were identified from those most frequently represented within TriNetX and placed within the R-ISD cohort. This cohort consisted of medications including abatacept, adalimumab, basiliximab, and others listed in Table 1. Receptor-mediated therapies exclusively used in short-term induction therapy or rejection treatment, including alemtuzumab and antithymocyte globulin, were excluded in this analysis.

Additionally, corticosteroids were not included as independent exposure variables due to substantial variability in dosing, duration, and indication across transplant and non-transplant populations. Their frequent intermittent use for induction therapy, rejection treatment, or acute disease flares limited reliable longitudinal exposure assessment within the TriNetX database. Furthermore, in transplant recipients, corticosteroids are commonly co-administered alongside multiple maintenance immunosuppressive agents and are often adjusted dynamically over time based on clinical status. As a result, accurately quantifying cumulative steroid exposure and isolating its independent contribution to malignancy risk was not feasible within the constraints of this retrospective database study. We therefore focused our analysis on maintenance immunosuppressive agents that more consistently define long-term immunosuppressive strategy, while acknowledging background corticosteroid exposure as a potential residual confounder.

The experimental cohort evaluating T-ISDs was defined to be mutually exclusive. Patients were excluded if they had ever received a C-ISD or R-ISD at any time. Eligible individuals were required to undergo an organ transplant (Table 1) and to have developed a new malignancy occurring at least three years after transplantation. To avoid evaluating risk of cancer recurrence, individuals with any documented neoplasia prior to treatment were excluded. Because T-ISDs are initiated immediately after transplant surgery, the index event for this cohort was defined as the date of transplantation. In contrast, the index event for the C-ISD and R-ISD cohorts was defined as each patient’s first recorded exposure to their respective ISD.

The C-ISD and R-ISD comparison groups were constructed similarly, differing only in the medications included. Patients in these two groups were excluded if they had ever received an organ transplant or any dose of a T-ISD. To ensure adequate exposure and follow-up, individuals in these two non-transplant cohorts were required to have at least 24 documented encounters involving their respective ISDs. This threshold was chosen to best compare T-ISDs, which patients will be taking their whole lives, to either C-ISDs or R-ISDs, where we desired for patients to take a given ISD for approximately two years, to adequately compare cancer risk. Patients with any history of neoplasia prior to the start of ISD therapy were excluded. As noted, the index event for both groups was the first ISD exposure meeting these criteria.

Following cohort construction, the T-ISD cohort was propensity score matched to each non-transplant comparison cohort for the malignancy analyses. Matching was performed using the native TriNetX algorithm, which applies 1:1 greedy nearest-neighbor matching without replacement. Variables selected for matching included demographic factors (age at index, sex, and race/ethnicity) and comorbidities associated with either increased malignancy risk or increased likelihood of receiving immunosuppression or transplantation. These included essential hypertension, heart failure, cardiomyopathy, diabetes mellitus, infectious mononucleosis, HIV infection, chronic kidney disease, renal dialysis, chronic viral hepatitis, body mass index, and a range of chronic gastrointestinal and hepatobiliary diseases. Table 2 provides an example of the complete pre- and post-matching variable set for the TAC cohort.

Post-matching analyses compared malignancy risk between the T-ISD cohort and each non-transplant cohort. Malignancy outcomes included in the initial analyses are listed in Table 3. Outcomes were indexed from one day to three years after the relevant index event. As noted, the index was the transplant date for the T-ISD cohort and the first qualifying ISD encounter for the non-transplant cohorts. Hazard ratios and 95% confidence intervals were computed for each matched comparison.

Subsequent analyses examined malignancy risk associated with each individual T-ISD. Individual cohorts for TAC, CY, MMF, and RAPA were constructed following the same methodology as the overall T-ISD cohort, with one key modification: each experimental cohort was mutually exclusive such that patients could only have received one of the four transplant-related ISDs and no exposure to the other three. The C-ISD and R-ISD cohorts from the initial analyses were retained as comparison groups. Matching procedures and the analytic time window (one day to three years following the index event) were identical to those used in the broader analysis.

For each individual T-ISD, malignancy outcomes were initially pooled using the full composite list from Table 3, mirroring the structure of the initial study. These aggregated outcomes were used to generate overall hazard ratios and confidence intervals before proceeding to more granular malignancy-specific analyses. While the grouped outcomes provide a global estimate of overall malignancy risk associated with each T-ISD relative to the two non-transplant immunosuppressive categories, they do not allow determination of which specific cancer types or anatomic sites may be differentially affected.

Therefore, individual T-ISDs were further evaluated using a set of site-specific malignancy outcomes (Table 4). Comparative analyses were performed between each T-ISD cohort and each non-transplant comparator cohort. Hazard ratios and 95% confidence intervals were calculated for each malignancy type, offering a more detailed profile of cancer risk potentially attributable to specific ISDs.

Overall, this multi-stage cohort design, combined with rigorous exclusion criteria, index standardization, and propensity score matching, enabled a structured comparison of malignancy risks across multiple classes of immunosuppressive medications used either in transplant recipients or in non-transplant autoimmune disease populations.

## 3. Results

The initial study comparing all four T-ISDs (TAC, CY, RAPA, MMF) to either control group (C-ISDs or R-ISDs) had varying-sized cohorts. The T-ISD cohort prior to propensity matching had 110,675 patients, while the C-ISD and R-ISD cohorts had 36,605 and 44,567 patients, respectively. Isolating the number of patients who received either TAC, CY, RAPA, or MMF to determine isolated malignancy risk of each T-ISD resulted in 13,925, 2992, 917, and 3127 patients, respectively.

Patients from the T-ISD cohort (patients who received either TAC, CY, MMF, or RAPA) were then compared to either the C-ISD or R-ISD cohorts that had not received any of the T-ISDs. Then similar studies were performed comparing individual T-ISDs to either C-ISD or R-ISD cohorts.

To identify overall trends between cohorts, the risk of malignancy associated with the T-ISD cohort was compared to both the C-ISD and R-ISD cohorts. This analysis identified that T-ISDs are associated with increased risk of cancer compared to both non-transplant C-ISDs (n: 29,748, HR: 1.271, CI: 1.195 to 1.351) and R-ISDs (n: 31,704, HR: 2.616, CI: 2.427 to 2.820).

To determine if isolated T-ISDs carry equal risk of malignancy compared to the other cohorts, individual analyses were obtained for individual ISDs. Additionally, to determine if certain malignancies were more commonly implicated, individual ISD analyses were performed for malignancies within different organ systems. The results of each analysis for an individual T-ISD compared to either C-ISD or R-ISD are found in Table 5 and Table 6.

When analyzing the risk of malignancy by comparing individual T-ISDs to C-ISDs, many interesting trends arose. In the analyses with outcomes of “ALL” malignancies throughout the body, only TAC and CY had significant results showing an associated increased risk of malignancy (TAC: HR = 1.354, CI = 1.228 to 1.492, CY: HR = 1.234, CI = 1.007 to 1.512). After performing isolated analyses of each individual organ system, TAC was associated with increased risk of malignancy compared to C-ISDs in liver cancer (HR: 9.667, 95% CI: 6.534 to 14.326), kidney cancer (HR: 3.794, 95% CI: 2.024 to 7.112), and lung cancer (HR: 1.953, 95% CI: 1.247 to 3.056). CY had an increased risk of malignancy in liver cancer (HR: 7.983, 95% CI: 2.792 to 22.822), kidney cancer (HR: 4.513, 95% CI: 1.484 to 13.725), and breast cancer (HR: 2.793, 95% CI: 1.138 to 6.856). RAPA did not show statistical significance. MMF demonstrated a correlated increased risk of malignancy in liver cancer (HR: 4.207, 95% CI: 1.539 to 11.501) and kidney cancer (HR: 4.953, 95% CI: 1.995 to 12.298).

Of the analyses performed for lymphoma, TAC, RAPA, and CY did not show any associated increased risk of malignancy compared to C-ISDs. However, MMF had a related decreased risk of malignancy for lymphoma compared to the C-ISDs (HR: 0.371, 95% CI: 0.183 to 0.754). The analyses performed for leukemia showed that all four T-ISDs had an association of decreased risk of malignant neoplasia compared to C-ISDs.

The results obtained from the analyses for individual T-ISDs compared to non-transplant R-ISDs displayed different trends from the non-transplant C-ISD study. The individual T-ISDs showed relative increased risk of malignant neoplasia with the analyses involving “ALL” malignancies as compared to R-ISDs (Table 6).

TAC was associated with increased risk of malignancy with the individual organ system malignancy types compared to R-ISDs except for liver cancer (confidence interval too large to make adequate connection), male reproductive cancer (HR: 1.082, 95% CI: 0.743 to 1.576), breast cancer (HR: 1.148, 95% CI: 0.682 to 1.932), and brain cancer (HR: 1.017, 95% CI: 0.272 to 3.797). CY showed a relatively increased risk of malignancy with all malignancy types except for lung cancer (HR: 2.268, 95% CI: 0.663 to 7.759), breast cancer (HR: 1.896, 95% CI: 0.912 to 3.938), and brain cancer (HR: 1.317, 95% CI: 0.082 to 21.133). RAPA demonstrated a relative increased risk of malignant neoplasia for the cancers excluding leukemia (HR: 0.755, 95% CI: 0.755 to 18.601), female reproductive cancer (HR: 0.384 to 35.611), male reproductive cancer (HR: 0.414, 95% CI: 0.112 to 1.528), kidney cancer (HR: 2.646, 95% CI: 0.484 to 14.462), and breast cancer (HR: 1.641, 95% CI: 0.44 to 6.124).

## 4. Discussion

Immunosuppressive drugs (ISDs) are essential for preventing organ rejection after transplantation and for treating autoimmune and inflammatory diseases. However, chronic immunosuppression has been associated with increased malignancy risk, largely due to impaired immunosurveillance and decreased clearance of oncogenic viruses [27,28]. Specifically, natural killer (NK) cells play a central role in immune surveillance by identifying and eliminating transformed or virus-infected cells before they can establish clinically detectable disease [29]. This cytotoxic function represents a key component of the body’s early defense against malignancy [29]. Immunosuppressive therapies used in both transplant and non-transplant settings may impair NK cell number and function to varying degrees, thereby reducing this tumor surveillance capacity [30,31]. While NK cell activity was not directly measured in this study, its suppression provides a biologically plausible mechanism that may partially underline the observed associations between immunosuppressive drug exposure and increased malignancy risk.

While prior studies have evaluated cancer risk in specific ISDs or compared transplant immunosuppressive drugs (T-ISDs) to the general population, few large-scale analyses have compared malignancy risk of T-ISDs to either non-transplant cell-mediated (C-ISD) or receptor-mediated ISDs (R-ISD) [21]. A more detailed comparison across these classes is critical for understanding long-term toxicity and guiding individualized treatment decisions.

In this retrospective cohort study, T-ISDs as a group were associated with higher malignancy risk than both C-ISDs and R-ISDs. After establishing this overall association, individual transplant agents tacrolimus (TAC), cyclosporine (CY), mycophenolate mofetil (MMF), and sirolimus (RAPA) were evaluated for overall and site-specific cancer risks.

Among T-ISDs, TAC and CY demonstrated the greatest relative malignancy risk, although the magnitude varied by organ system. RAPA showed mixed results, with no significant difference compared to C-ISDs but higher risk relative to R-ISDs. MMF followed a similar pattern but had the lowest associated malignancy risk among T-ISDs.

Compared with C-ISDs, TAC and CY each demonstrated increased risk for three of nine evaluated cancers. TAC was associated with elevated liver, kidney, and lung cancer risk, whereas CY was associated with liver, kidney, and breast cancer. These findings are plausible, as both are calcineurin inhibitors. TAC binds FK-binding protein to inhibit calcineurin, while CY binds cyclophilin-1 to form an inhibitory complex that suppresses T-cell activation [32]. MMF showed increased risk in two cancers (liver and kidney), and RAPA in one (lung). However, the smaller RAPA cohort may have limited statistical power. Overall, TAC and CY carried the greatest malignancy risk relative to C-ISDs.

MMF demonstrated comparatively favorable outcomes, with lower hazard ratios and narrower 95% confidence intervals across cancer sites. MMF inhibits inosine-5′-monophosphate reductase, suppressing T- and B-cell proliferation [33]. Emerging evidence suggests possible anti-tumor effects, including inhibition of cancer colony formation in osteosarcoma and gastrointestinal tumors [34,35]. Notably, MMF was the only T-ISD associated with decreased lymphoma risk relative to C-ISDs, further supporting a potential anti-neoplastic effect.

When compared to R-ISDs, trends were similar but more pronounced. TAC demonstrated elevated risk in five of nine cancers, including lymphoma, leukemia, female reproductive, kidney, and lung cancers. CY showed increased risk in five cancers: lymphoma, leukemia, female and male reproductive, and kidney. RAPA and MMF each showed increased risk for two cancers: RAPA for lymphoma and lung, and MMF for lymphoma and kidney. Cancer types in which at least half of the T-ISDs showed elevated risk included lymphoma, leukemia, female reproductive, kidney, and lung malignancies (Table 6).

Overall, hazard ratios and 95% confidence intervals were higher in T-ISD versus R-ISD analyses than in T-ISD versus C-ISD comparisons, suggesting a greater relative malignancy risk of T-ISDs compared to R-ISDs. For example, TAC showed increased risk in six cancers versus R-ISDs but only three versus C-ISDs. These findings support the perception that R-ISDs may carry a more favorable malignancy profile.

R-ISDs are increasingly favored due to their targeted mechanisms and malignancy profiles [36]. They are used in autoimmune and inflammatory diseases and in oncology for cancer-targeting capabilities [37,38]. Their application in transplantation, particularly renal transplantation, has shown promising outcomes [39,40]. However, high cost remains a significant barrier to widespread long-term use of monoclonal and polyclonal antibody therapies [41]. Additional limitations include infusion reactions, increased infection risks, neurologic symptoms, and gastrointestinal side effects [36]. Many require intravenous administration, further limiting convenience. Although their role in transplant medicine may expand, these practical challenges remain substantial.

In contrast, MMF is widely available, relatively cost-effective, and demonstrated one of the lowest malignancy risk profiles among T-ISDs. These findings suggest that in transplant recipients at higher baseline cancer risk, MMF monotherapy compared to TAC monotherapy may be associated with lower malignancy risk. While causality cannot be established due to the retrospective design, general trends are informative. TAC is commonly paired with MMF because of its strong anti-rejection efficacy, and studies show that TAC combined with MMF reduces rejection compared to TAC alone [42,43]. However, MMF monotherapy, though not routine, may offer a safer long-term option in selected patients by reducing nephrotoxicity and possibly malignancy risk [44].

Several limitations should be noted. Patients likely received ISDs for variable durations, which may have influenced cancer incidence. Although a minimum exposure threshold was applied, cumulative exposure differences may not have been fully captured. Analyses involving R-ISDs excluded transplant patients previously exposed to R-ISDs, reducing sample size, particularly since many patients receive R-ISDs as induction therapy before transitioning to maintenance regimens. Future studies should evaluate how induction therapy with R-ISDs influences long-term malignancy risk when followed by T-ISDs.

Although propensity score matching was performed for multiple demographic and comorbid variables, residual confounding from underlying disease processes may still exist. Certain chronic inflammatory or autoimmune conditions requiring immunosuppressive therapy may independently increase baseline malignancy risk and could not be fully accounted for within the limitations of the TriNetX database. Additionally, clinically relevant infections such as Epstein–Barr virus (EBV) viremia or Cytomegalovirus were difficult to reliably assess due to variability in laboratory reporting and coding practices across participating healthcare systems as well as within the TriNetX database itself. We incorporated relevant infectious diagnosis codes into the propensity score matching to account for the potential contribution of specific infections to the development of malignancy. Future studies should incorporate specific infectious agents, including EBV, BK virus, and CMV, as explicit variables to better elucidate the relationship between immunosuppressive drug classes, opportunistic infections, and subsequent malignancy risks.

Additionally, some subgroup analyses included small numbers of malignancy events, with certain cohorts reporting ten or fewer cases. To isolate malignancy risk attributable to each T-ISD, exposure groups were restricted to exclusive monotherapy use. While this improved attribution to individual agents, it reduced cohort size and excluded patients treated with contemporary multi-agent regimens. Thus, hazard ratios primarily reflect monotherapy effects rather than real-world combination therapy. Dosing intensity and target levels were not captured or analyzed in this study, as our primary objective was to evaluate overall exposure to specific immunosuppressive agents rather than dose-dependent effects. Future studies should evaluate dose-dependent parameters within immunosuppressive regimens to better characterize the relationship between dosing intensity and malignancy risk. Finally, the outcome window of one day to three years may have excluded late-onset cancers, potentially underestimating long-term malignancy risks.

In summary, T-ISDs were associated with higher malignancy risk compared to both C-ISDs and R-ISDs, with TAC and CY demonstrating the greatest relative risk. MMF exhibited the most favorable malignancy profile among transplant agents and may represent a lower-risk alternative in selected patients. Although causality cannot be inferred, these findings provide clinically meaningful insights into comparative malignancy risks and may help inform individualized immunosuppressive strategies.

## 5. Conclusions

In the T-ISD combined analysis, T-ISDs were associated with higher risk of overall malignancy than both C-ISDs and R-ISDs, with risk being greatest relative to R-ISDs. Among the four T-ISDs studied (TAC, CY, MMF, and RAPA), TAC monotherapy showed the highest overall cancer risk, while MMF and RAPA monotherapies had the lowest. MMF monotherapy demonstrated the most favorable and statistically significant malignancy profile, suggesting lower relative risk of malignant neoplasia as compared to other ISDs. Cancers where at least half of the T-ISDs showed elevated risk compared to C-ISDs included liver, lung, and kidney cancers. Against R-ISDs, the most affected were lymphoma, leukemia, female reproductive, kidney, and lung cancers.

These findings provide important guidance to clinicians about comparative malignancy risks of ISDs used in transplantations versus those used for other conditions in non-transplant patients. They highlight the need to reconsider long-term immunosuppressive strategies or targeted surveillance to reduce cancer risks in transplant patients. Given its low malignancy risk, well-known cost-effectiveness and tolerable safety profile, MMF monotherapy or increased use in combination warrants further investigation as a primary option for maintenance immunosuppression. Future studies should confirm these findings and identify optimal regimens for balancing treatment efficacy with reduced malignancy risk.

## Figures and Tables

**Table 1:** Three initial cohort lists of ISDs and characteristics (ICD-10, CPT, or VA codes) that patients in their respective cohort either had (+) or did not have (-).

ICD-10 Codes per Cohort (Initial Study)		
Transplant ISDs	Non-Transplant Cell-Mediated ISDs	Non-Transplant Receptor-Mediated ISDs
(+) Tacrolimus (42316)	(+) Methotrexate (6851)	(+) Abatacept (614391)
(+) Cyclosporine (3008)	(+) Azathioprine (1256)	(+) Adalimumab (327361)
(+) Mycophenolate Mofetil (68149, 7145, and 265323)	(+) Cyclophosphamide (3002)	(+) Etanercept (214555)
(+) Rapamycin (35302)	(+) Hydroxyurea (5552)	(+) Basiliximab (196102)
(-) Neoplasia (C00-D49)	(+) Capecitabine (194000)	(+) Infliximab (191831)
(-) C-ISDs or R-ISDs	(+) Leflunomide (27169)	(+) Omalizumab (302379)
(+) Kidney Transplant Status (Z94.0)	(-) Neoplasia (C00-D49)	(+) Tocilizumab (612865)
(+) Heart Transplant Status (Z94.1)	(-) R-ISDs	(+) Certolizumab Pegol (709271)
(+) Lung Transplant Status (Z94.2)	(-) Kidney Transplant Status (Z94.0)	(+) Vedolizumab (1538097)
(+) Heart and Lungs Transplant Status (Z94.3)	(-) Heart Transplant Status (Z94.1)	(-) Neoplasia (C00-D49)
(+) Liver Transplant Status (Z94.4)	(-) Lung Transplant Status (Z94.2)	(-) T-ISDs
(+) Transplantation (02Y, 0BY, 0FY, 0TY)	(-) Heart and Lungs Transplant Status (Z94.3)	(-) Kidney Transplant Status (Z94.0)
	(-) Liver Transplant Status (Z94.4)	(-) Heart Transplant Status (Z94.1)
	(-) Transplantation (02Y, 0BY, 0FY, 0TY)	(-) Lung Transplant Status (Z94.2)
		(-) Heart and Lungs Transplant Status (Z94.3)
		(-) Liver Transplant Status (Z94.4)
		(-) Transplantation (02Y, 0BY, 0FY, 0TY)

**Table 2:** Propensity-matched patients with a list of baseline characteristics according to given demographics and diagnoses of the T-ISD TAC cohort compared to the non-transplant R-ISDs.

	Before Propensity Match					After Propensity Match				
Demographic	TAC	n	R-ISDs	n	*p*-Value	TAC	n	R-ISDs	n	*p*-Value
Age at Index	50.5 ± 20.3	13,761	43 ± 19.7	42,781	<0.0001	49.5 ± 20.6	11,571	50.8 ± 19	11,571	<0.0001
Male	54.76%	7678	38.01%	16,454	<0.0001	52.03%	6037	49.61%	5802	0.0003
Female	42.31%	5705	60.22%	25,559	<0.0001	45.43%	5240	47.56%	5482	0.0015
Hispanic or Latino	9.05%	1148	8.57%	3582	0.0874	9.25%	1011	9.37%	958	0.7639
Not Hispanic or Latino	63.22%	8245	74.75%	32,927	<0.0001	65.48%	7462	65.02%	7375	0.472
White	64.80%	8655	73.76%	31,189	<0.0001	66.85%	7576	66.06%	7572	0.2108
Black or African American	14.92%	2190	10.31%	4597	<0.0001	13.47%	1629	13.51%	1580	0.9373
Asian	4.06%	559	2.70%	1192	<0.0001	3.79%	429	3.86%	463	0.7794
American Indian or Alaska Native	0.43%	40	0.51%	187	0.2682	0.43%	37	0.37%	40	0.4572
Native Hawaiian or Other Pacific Islander	0.41%	51	0.23%	98	0.0005	0.42%	42	0.39%	45	0.75
Diagnosis	TAC	n	R-ISDs	n	*p*-value	TAC	n	R-ISDs	n	*p*-value
Diseases of Liver	12.07%	1473	3.40%	1371	<0.0001	8.56%	913	8.55%	903	0.9808
Chronic Kidney Disease	12.81%	1817	1.91%	753	<0.0001	6.82%	729	6.03%	628	0.0172
Essential Hypertension	12.98%	1785	15.47%	6297	<0.0001	11.42%	1278	10.97%	1255	0.2967
Diabetes Mellitus	9.40%	1302	6.56%	2640	<0.0001	7.54%	814	7.77%	820	0.5114
Diseases of Esophagus, Stomach and Duodenum	7.30%	1049	18.95%	7728	<0.0001	7.28%	843	6.93%	795	0.3206
Disorders of Gallbladder, Biliary Tract, and Pancreas	4.97%	662	3.38%	1383	<0.0001	4.08%	457	3.86%	442	0.409
Heart Failure	3.57%	490	1.60%	635	<0.0001	2.66%	286	2.41%	255	0.2316
BMI	3.29%	451	6.97%	2799	<0.0001	3.11%	350	3.08%	333	0.9074
Chronic Viral Hepatitis	2.43%	261	0.42%	186	<0.0001	1.33%	129	1.22%	116	0.472
Renal Dialysis	0.41%	50	0.20%	10	<0.0001	0.16%	16	0.09%	10	0.1303
HIV	0.16%	30	0.08%	35	0.0087	0.12%	18	0.14%	14	0.5772
Infectious Mononucleosis	0.12%	17	0.22%	90	0.0219	0.11%	14	0.18%	13	0.157

**Table 3:** Grouped ICD-10 codes for malignancy risk outcomes used in the initial comparison of the T-ISDs to either non-transplant C-ISDs or R-ISDs.

ICD-10 Codes for Malignancy Risk Outcomes	
Name	ICD-10 Code
Melanoma and other malignant neoplasms of skin	C43–C44
Malignant neoplasms of digestive organs	C15–C26
Malignant neoplasms of lymphoid, hematopoietic and related tissue	C81–C96
Malignant neoplasms of urinary tract	C64–C68
Malignant neoplasms of male genital organs	C60–C63
Malignant neoplasms of respiratory and intrathoracic organs	C30–C39
Malignant neoplasms of breast	C50–C50
Malignant neoplasms of lip, oral cavity and pharynx	C00–C14
Malignant neoplasms of female genital organs	C51–C58
Malignant neoplasms of thyroid and other endocrine glands	C73–C75
Malignant neoplasms of eye, brain and other parts of CNS	C69–C72
Malignant neoplasms of bone and articular cartilage	C40–C41
Malignant neuroendocrine tumors	C7A–C7A
Malignant neoplasms of mesothelial and soft tissue	C45–C49

**Table 4:** Malignancy outcomes and their associated ICD-10 codes chosen for individual T-ISDs vs. either non-transplant C-ISDs or R-ISDs analyses.

Individual T-ISD Study ICD-10 Codes for Malignancy Risk Outcomes	
Name	ICD-10 Code
1. Liver Cancer	C22.0, C22.2, C22.8
2. Lymphoma	C81–C86
3. Leukemia	C90–C95
4. Malignancies of Female Reproductive System	C51–C58
5. Malignancies of Male Reproductive System	C60–C63
6. Malignancies of the Kidney	C64
7. Malignancies of the Lung	C34
8. Malignancies of Breast	C50
9. Malignancies of Brain and CNS	C71–C72

**Table 5:** Table showing hazard ratios (HRs) and 95% confidence intervals (95% CIs) for risks of different types of malignant neoplasia for individual T-ISDs compared to non-transplant C-ISDs. “n” indicates initial sample size of cohort comparison after propensity matching. * indicates the non-transplant C-ISD cohort had less than 10 patients with a given outcome while ** signifies the cohort had zero patients with the given outcome of malignant neoplasia.

Individual T-ISDs vs. C-ISDs								
Cancer	ICD-10	Drug	n	Cancer Cases	HR	95% CI		
ALL	C00–C26 C30–C41 C43–C58 C60–C96	TAC	9846	832	1.354	1.228	to	1.492
		MMF	2621	165	1.106	0.901	to	1.357
		CY	1801	184	1.234	1.007	to	1.512
		RAPA	753	75	1.335	0.963	to	1.852
Liver	C22.0 C22.2 C22.8	TAC	10,432	221	9.667	6.523	to	14.326
		MMF *	2806	16	4.207	1.539	to	11.501
		CY *	1870	27	7.983	2.792	to	22.822
		RAPA **	748	12	N/A	N/A	to	N/A
Lymphoma	C81–C86	TAC	10,432	85	0.813	0.617	to	1.072
		MMF	2806	≤10	0.371	0.183	to	0.754
		CY	1870	21	1.216	0.664	to	2.228
		RAPA *	775	≤10	2.953	0.925	to	9.425
Leukemia	C90–C95	TAC	10,433	52	0.153	0.114	to	0.204
		MMF	2806	12	0.117	0.065	to	0.212
		CY	1870	20	0.353	0.214	to	0.582
		RAPA	775	≤10	0.171	0.06	to	0.491
Female Repro	C51–C58	TAC	10,433	26	0.872	0.527	to	1.446
		MMF	2806	≤10	0.728	0.286	to	1.854
		CY *	1870	≤10	0.712	0.258	to	1.96
		RAPA *	775	≤10	3.519	0.366	to	33.859
Male Repro	C60–C63	TAC	9129	45	0.68	0.474	to	0.976
		MMF	2384	14	0.726	0.38	to	1.387
		CY	1638	17	0.849	0.461	to	1.567
		RAPA	669	≤10	0.331	0.092	to	1.187
Kidney	C64	TAC	8796	39	3.794	2.024	to	7.112
		MMF *	2427	21	4.953	1.995	to	12.298
		CY *	1756	14	4.513	1.484	to	13.725
		RAPA *	663	≤10	1.873	0.312	to	11.234
Lung	C34	TAC	8928	48	1.953	1.247	to	3.056
		MMF *	2259	≤10	1.973	0.681	to	5.715
		CY *	1749	≤10	0.684	0.206	to	2.274
		RAPA *	690	≤10	10.489	1.328	to	82.847
Breast	C50	TAC	9017	24	0.864	0.515	to	1.45
		MMF	3132	≤10	0.585	0.224	to	1.523
		CY *	1733	15	2.793	1.138	to	6.856
		RAPA *	684	≤10	2.203	0.526	to	9.234
Brain	C71–C72	TAC *	9241	≤10	1.017	0.272	to	3.797
		MMF	2428	0	N/A	N/A	to	N/A
		CY *	1760	≤10	1.337	0.083	to	21.47
		RAPA **	734	≤10	N/A	N/A	to	N/A

**Table 6:** Table showing hazard ratios (HRs) and 95% confidence intervals (95% CIs) for risks of different types of malignant neoplasia for individual T-ISDs compared to non-transplant R-ISDs. “n” indicates sample size of cohort comparison after propensity-matching. * indicates the non-transplant R-ISD cohort had less than 10 patients with a given outcome while ** signifies the cohort had zero patients with the given outcome of malignant neoplasia.

Individual T-ISDs vs. R-ISDs								
Cancer	ICD-10	Drug	n	Cancer Cases	HR	95% CI		
ALL	C00–C26 C30–C41 C43–C58 C60–C96	TAC	11,571	1020	2.755	2.472	to	3.071
		MMF	2689	180	1.721	1.384	to	2.139
		CY	2371	252	2.55	2.062	to	3.154
		RAPA	834	82	2.181	1.526	to	3.115
Liver	C22.0 C22.2 C22.8	TAC *	11,453	218	90.594	28.99	to	283.116
		MMF **	2747	15	N/A	N/A	to	N/A
		CY **	2335	25	N/A	N/A	to	N/A
		RAPA **	818	13	N/A	N/A	to	N/A
Lymphoma	C81–C86	TAC	12,054	118	4.702	3.165	to	6.985
		MMF	2849	14	2.807	1.13	to	6.976
		CY *	2432	24	5.842	2.227	to	15.315
		RAPA *	860	12	7.332	1.639	to	32.79
Leukemia	C90–C95	TAC	12,053	68	2.484	1.645	to	3.751
		MMF *	2848	13	1.969	0.84	to	4.617
		CY *	2432	25	3.04	1.459	to	6.335
		RAPA *	860	≤10	3.746	0.755	to	18.601
Female Repro	C51–C58	TAC	12,053	32	1.816	1.055	to	3.127
		MMF *	2848	≤10	2.231	0.652	to	7.641
		CY *	2432	≤10	3.7	1.001	to	13.681
		RAPA *	860	≤10	3.697	0.384	to	35.611
Male Repro	C60–C63	TAC	11,014	50	1.082	0.743	to	1.576
		MMF	2587	13	0.827	0.418	to	1.635
		CY	2261	30	2.752	1.458	to	5.193
		RAPA *	792	≤10	0.414	0.112	to	1.528
Kidney	C64	TAC	10,233	44	3.434	1.937	to	6.089
		MMF *	2432	18	4.157	1.646	to	10.497
		CY *	2176	21	3.884	1.65	to	9.144
		RAPA *	708	≤10	2.646	0.484	to	14.462
Lung	C34	TAC	10,619	54	3.435	2.055	to	5.742
		MMF *	2471	≤10	1.94	0.67	to	5.619
		CY *	2324	≤10	2.268	0.663	to	7.759
		RAPA *	804	≤10	10.906	1.38	to	86.175
Breast	C50	TAC	10,497	27	1.148	0.682	to	1.932
		MMF	3376	≤10	0.768	0.339	to	1.741
		CY	2304	18	1.896	0.912	to	3.938
		RAPA *	750	≤10	1.641	0.44	to	6.124
Brain	C71–C72	TAC *	11,089	≤10	1.017	0.272	to	3.797
		MMF *	2571	≤10	0.77	0.069	to	8.585
		CY *	2192	≤10	1.317	0.082	to	21.133
		RAPA **	769	≤10	N/A	N/A	to	N/A

## Data Availability

The original contributions presented in this study are included in the article. Further inquiries can be directed to the corresponding author.

## Abbreviations

The following abbreviations are used in this manuscript:

T-ISDs	Traditionally Used Transplant Immunosuppressants
R-ISDs	Receptor-Mediated Non-Transplant Immunosuppressants
C-ISDs	Cell-Mediated Non-Transplant Immunosuppressants

## References

[1] Allison A.C. (2000). Immunosuppressive drugs: The first 50 years and a glance forward. Immunopharmacology.

[2] Schwartz R., Dameshek W., Donovan J. (1960). THE EFFECTS OF 6-MERCAPTOPURINE ON HOMOGRAFT REACTIONS *. J. Clin. Investig..

[3] Schwartz R., Dameshek W. (1959). Drug-induced immunological tolerance. Nature.

[4] Murray J.E., Merrill J.P., Harrison J.H., Wilson R.E., Dammin G.J. (1984). Prolonged survival of Human-Kidney homografts by immunosuppressive drug therapy. Ann. Plast. Surg..

[5] Mortezavi M., Martin D.A., Schulze-Koops H. (2022). After 25 years of drug development, do we know JAK?. RMD Open.

[6] Wu N., Lee Y.-C., Shah N., Harrison D.J. (2014). Cost of Biologics per Treated Patient across Immune-mediated Inflammatory Disease Indications in a Pharmacy Benefit Management Setting: A Retrospective Cohort Study. Clin. Ther..

[7] Hariharan S., Johnson C.P., Bresnahan B.A., Taranto S.E., McIntosh M.J., Stablein D. (2000). Improved Graft Survival after Renal Transplantation in the United States, 1988 to 1996. N. Engl. J. Med..

[8] Wolfe R.A., Ashby V.B., Milford E.L., Ojo A.O., Ettenger R.E., Agodoa L.Y.C., Held P.J., Port F.K. (1999). Comparison of mortality in all patients on dialysis, patients on dialysis awaiting transplantation, and recipients of a first cadaveric transplant. N. Engl. J. Med..

[9] Cameron J.I., Whiteside C., Katz J., Devins G.M. (2000). Differences in quality of life across renal replacement therapies: A meta-analytic comparison. Am. J. Kidney Dis..

[10] Morren J., Li Y. (2019). Maintenance immunosuppression in myasthenia gravis, an update. J. Neurol. Sci..

[11] Page A., Fusil F., Cosset F.-L. (2021). Antigen-specific tolerance approach for rheumatoid arthritis: Past, present and future. Jt. Bone Spine.

[12] Durcan L., O’Dwyer T., Petri M. (2019). Management strategies and future directions for systemic lupus erythematosus in adults. Lancet.

[13] Gerriets V., Goyal A., Khaddour K. (2023). Tumor Necrosis Factor Inhibitors. StatPearls [Internet].

[14] McGlynn K.A., Petrick J.L., El-Serag H.B. (2020). Epidemiology of hepatocellular carcinoma. Hepatology.

[15] Stallone G., Infante B., Grandaliano G., Schena F.P., Gesualdo L. (2008). Kaposi’s sarcoma and mTOR: A crossroad between viral infection neoangiogenesis and immunosuppression. Transpl. Int..

[16] Andrés A. (2005). Cancer incidence after immunosuppressive treatment following kidney transplantation. Crit. Rev. Oncol./Hematol..

[17] Lutz J., Heemann U. (2003). Tumours after kidney transplantation. Curr. Opin. Urol..

[18] Snanoudj R., Durrbach A., Leblond V., Caillard S., De Ligny B.H., Noel C., Rondeau E., Moulin B., Mamzer-Bruneel M.-F., Lacroix C. (2003). Primary brain lymphomas after kidney transplantation: Presentation and outcome. Transplantation.

[19] Bilgi A., Gökulu Ş.G., İlgen O., Kulhan M., Kavurmacı S.A., Toz H., Terek M.C. (2021). Cervical dysplasia after renal transplantation: A retrospective cohort study. J. Turk. Soc. Obstet. Gynecol..

[20] Sherer B.A., Warrior K., Godlewski K., Hertl M., Olaitan O., Nehra A., Deane L.A. (2017). Prostate cancer in renal transplant recipients. Int. Braz. J. Urol..

[21] Cicalese L., Westra J.R., Walton Z.C., Kuo Y.-F. (2025). Immunosuppressant drug specific risk of malignancy after organ Transplantation: A Population-Based Analysis of Texas Medicare Beneficiaries. Cancers.

[22] Rodríguez-Perálvarez M., Colmenero J., González A., Gastaca M., Curell A., Caballero-Marcos A., Sánchez-Martínez A., Di Maira T., Herrero J.I., Almohalla C. (2022). Cumulative exposure to tacrolimus and incidence of cancer after liver transplantation. Am. J. Transplant..

[23] Ntobe-Bunkete B., Lemaitre F. (2024). Therapeutic drug monitoring in kidney and liver transplantation: Current advances and future directions. Expert Rev. Clin. Pharmacol..

[24] van den Born J., Meziyerh S., Vart P., Bakker S., Berger S., Florquin S., de Fijter J., Gomes-Neto A., Idu M., Pol R. (2024). Comparison of 2 Immunosuppression Minimization Strategies in Kidney Transplantation: The ALLEGRO Trial. Transplantation.

[25] Choi B., Ko Y., Kim J.-M., Kwon H.E., Kim Y.H., Shin S., Jung J.H., Kwon H. (2025). Tacrolimus–Sirolimus Combined Exposure and Acute Rejection in Kidney Transplant Recipients Undergoing Early Conversion to Sirolimus: A Multicenter Retrospective Cohort Threshold Analysis. J. Clin. Med..

[26] Davis J.S., Ferreira D., Paige E., Gedye C., Boyle M. (2020). Infectious complications of biological and small molecule targeted immunomodulatory therapies. Clin. Microbiol. Rev..

[27] Baan R.A., Stewart B.W., Straif K. (2019). Tumour Site Concordance and Mechanisms of Carcinogenesis.

[28] National Cancer Institute (2015). Risk Factors: Immunosuppression.

[29] Masmoudi D., Villalba M., Alix-Panabières C. (2025). Natural killer cells: The immune frontline against circulating tumor cells. J. Exp. Clin. Cancer Res..

[30] Portale F., Di Mitri D. (2023). NK Cells in Cancer: Mechanisms of Dysfunction and Therapeutic Potential. Int. J. Mol. Sci..

[31] Shang J., Hu S., Wang X. (2024). Targeting natural killer cells: From basic biology to clinical application in hematologic malignancies. Exp. Hematol. Oncol..

[32] Safarini O.A., Keshavamurthy C., Patel P. (2023). Calcineurin Inhibitors. StatPearls.

[33] Allison A.C. (2005). Mechanisms of Action of Mycophenolate Mofetil. Lupus.

[34] Klangjorhor J., Chaiyawat P., Teeyakasem P., Sirikaew N., Phanphaisarn A., Settakorn J., Lirdprapamongkol K., Yama S., Svasti J., Pruksakorn D. (2019). Mycophenolic Acid Is a Drug with the Potential to Be Repurposed for Suppressing Tumor Growth and Metastasis in Osteosarcoma Treatment. Int. J. Cancer.

[35] Leckel K., Beecken W., Jonas D., Oppermann E., Coman M.C., Beck K., Cinatl J., Hailer N.P., Auth M.K.H., Bechstein W.O. (2003). The immunosuppressive drug mycophenolate mofetil impairs the adhesion capacity of gastrointestinal tumour cells. Clin. Exp. Immunol..

[36] Eisenberg S. (2012). Biologic therapy. J. Infus. Nurs..

[37] Clark J.I., Weiner L.M. (1995). Biologic treatment of human cancer. Curr. Probl. Cancer.

[38] Papież M.A., Krzyściak W. (2021). Biological Therapies in the Treatment of Cancer—Update and New Directions. Int. J. Mol. Sci..

[39] Claeys E., Vermeire K. (2019). Immunosuppressive Drugs in Organ Transplantation to Prevent Allograft Rejection: Mode of Action and Side Effects. J. Immunol. Sci..

[40] Grenda R. (2014). Biologics in renal transplantation. Pediatr. Nephrol..

[41] Gary M.O. (2008). The value of biologics. Am. Health Drug Benefits.

[42] Gallon L., Dalal P., Shah G., Chhara D. (2010). Role of tacrolimus combination therapy with mycophenolate mofetil in the prevention of organ rejection in kidney transplant patients. Int. J. Nephrol. Renov. Dis..

[43] Boudjema K., Camus C., Saliba F., Calmus Y., Salamé E., Pageaux G., Ducerf C., Duvoux C., Mouchel C., Renault A. (2011). Reduced-Dose Tacrolimus with Mycophenolate Mofetil vs. Standard-Dose Tacrolimus in Liver Transplantation: A Randomized Study. Am. J. Transplant..

[44] Schmeding M., Kiessling A., Neuhaus R., Heidenhain C., Bahra M., Neuhaus P., Neumann U.P. (2011). Mycophenolate mofetil monotherapy in liver transplantation: 5-Year Follow-Up of a prospective randomized Trial. Transplantation.

